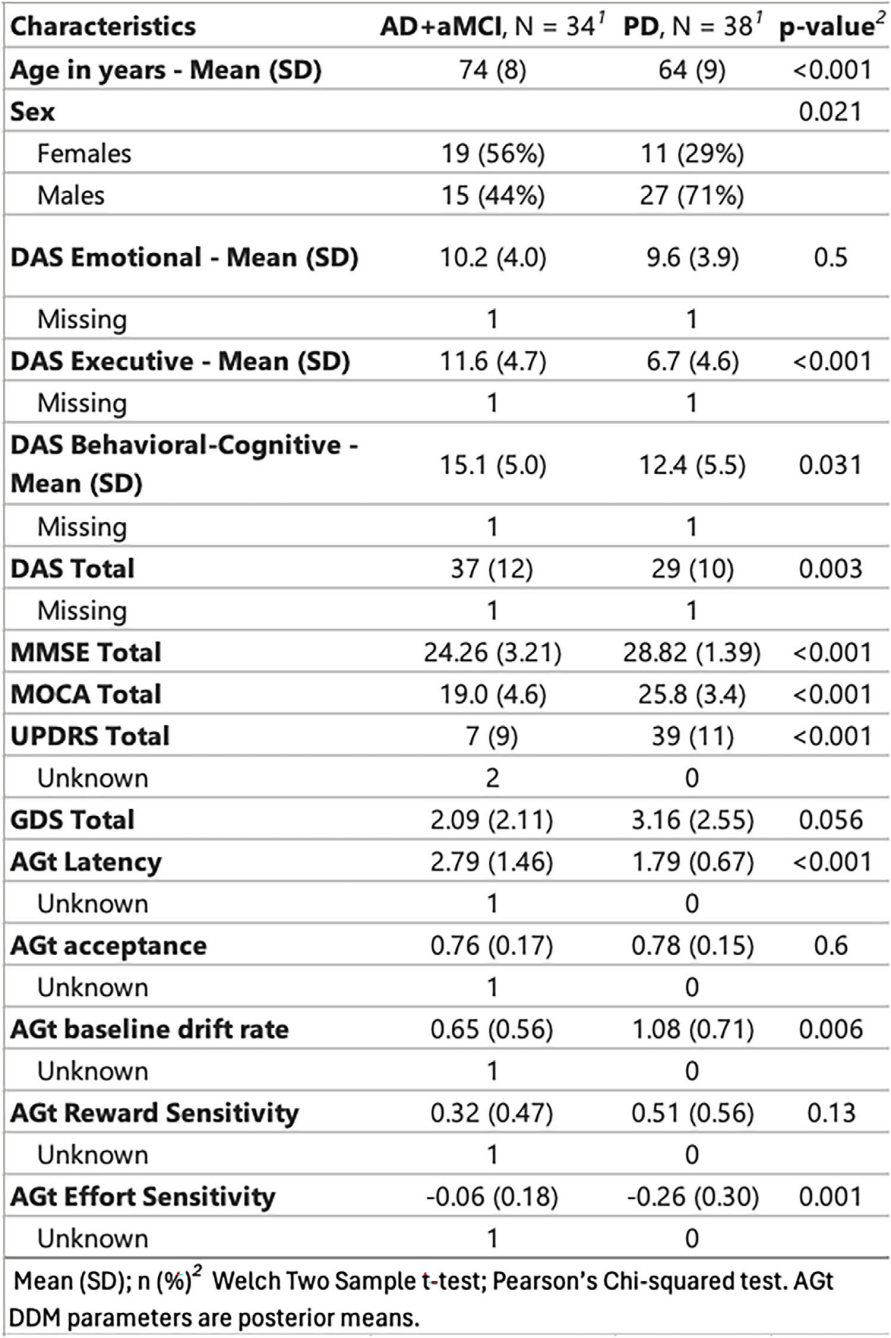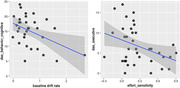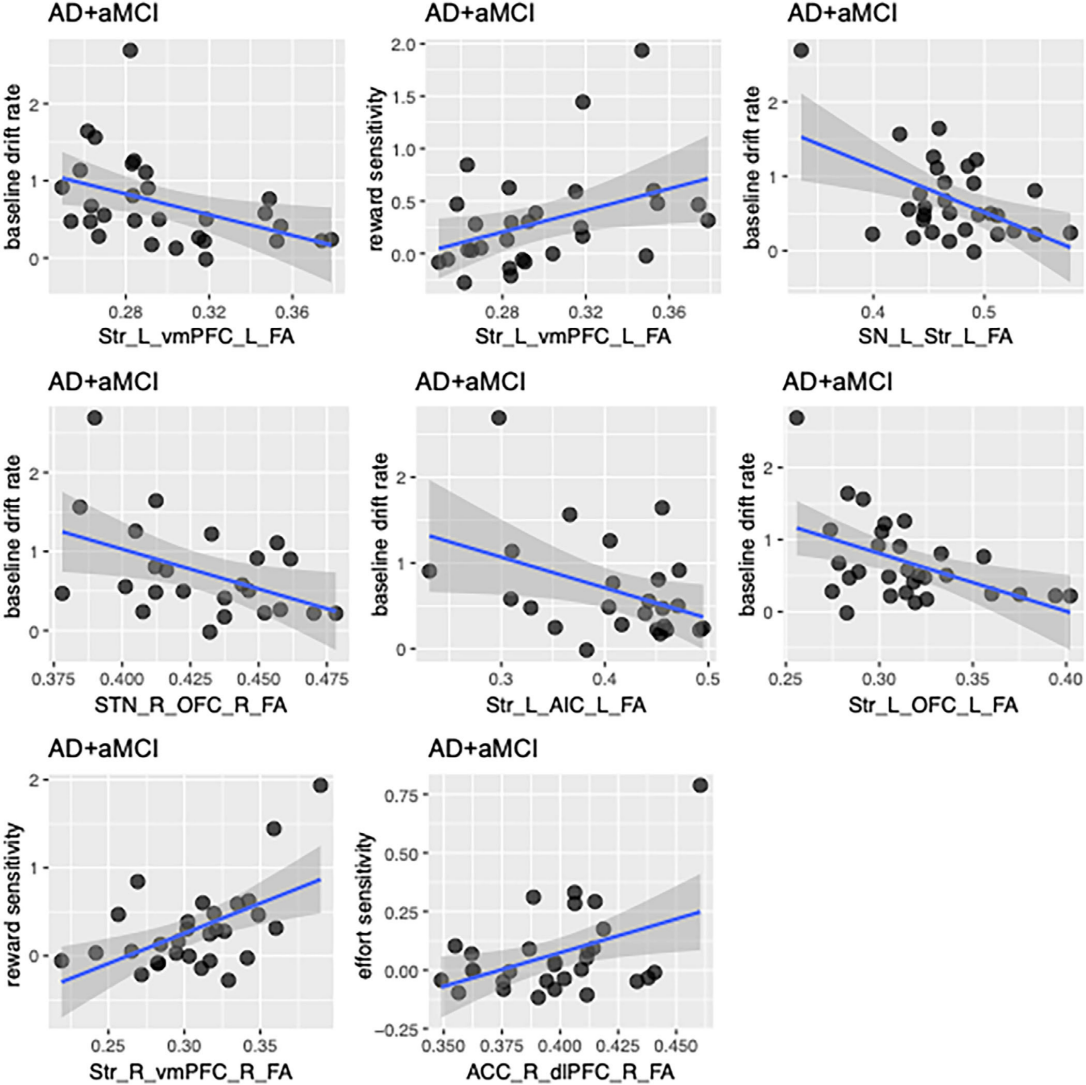# Effort‐based decision making and white matter tracts associated with dimensions of apathy in Alzheimer’s and Parkinson’s disease

**DOI:** 10.1002/alz.095204

**Published:** 2025-01-09

**Authors:** Yunglin Gazes, Seonjoo Lee, Zekai Jin, Bryan Chen, Edward D. Huey, Sarah Heibronner, Campbell Le Heron, Nora Vanegas

**Affiliations:** ^1^ Nathan Kline Institute, Orangeburg, NY USA; ^2^ Mailman School of Public Health, Columbia University, New York, NY USA; ^3^ Columbia University Irving Medical Center, New York, NY USA; ^4^ Brown University, Providence, RI USA; ^5^ Baylor College of Medicine, Houston, TX USA; ^6^ New Zealand Brain Research Institute, Christchurch New Zealand

## Abstract

**Background:**

Apathy is marked by diminished motivation and goal‐directed behavior, prevalent in neurodegenerative diseases like Alzheimer’s disease (AD) and Parkinson’s disease (PD). Effort‐based decision‐making paradigms (EBDM), which require choices between tasks of varying effort levels for varying rewards, are effective assessments of goal‐directed behavior. Using a transdiagnostic approach, we are examining the neurodegeneration of networks on apathy and EBDM.

**Method:**

We present preliminary results of a novel transdiagnostic study consisting of AD/amnestic Mild Cognitive Impairment (aMCI) and PD patients: 1) apathy is measured with Dimensional Apathy Scale (DAS), 2) an EBDM paradigm (Apple Gathering Task‐AGt) is used to estimate sensitivity to effort and reward, and microstructural integrity of relevant white matter tracts is estimated using fractional anisotropy [FA] from multi‐shell diffusion MRI. Probabilistic tractography in MRtrix toolbox was used to extract tracts connecting frontal‐subcortical regions. Using hierarchical Bayesian modeling and Drift Diffusion Model, we examined the associations among the apathy scale, behavioral performance, and brain health, covaried for age, MMSE, and UPDRS (PDs only).

**Result:**

Table 1 shows participant characteristics and group differences in study measures. Overall, AD/aMCI patients showed greater apathy and worse clinical scores for cognition (MMSE and MoCA) than PD patients. On AGt, AD/aMCI patients demonstrated longer latency and lower effort sensitivity. AD/aMCI patients showed lower baseline drift rate with higher scores for the behavioral‐cognitive dimension of DAS (95% credible interval [CrI]: ‐1.6 to ‐0.16, Figure 1). PDs showed less effort sensitivity with higher executive dimension of DAS (95% CrI: ‐2.8 to ‐0.23). In AD/aMCI, higher FA for the tract connecting right dorsal lateral prefrontal cortex to the striatum was associated with lower emotional apathy (95% CrI: ‐0.774, ‐0.048, Figure 2). In PDs, higher FA of Striatum‐Anterior Insula Cortex tract was associated with lower apathy in the executive dimension (95% CrI: ‐0.83, ‐0.161, Figure 2 upper left).

**Conclusion:**

Our results demonstrate distinctions in apathetic symptoms and behavioral performance for AD/aMCI and PD patients, and also in the role of different fronto‐subcortical white matter tracts in dimensions of apathetic symptoms. Future analysis in a larger cohort will directly test the suggested mediation effects of regional white matter quality on apathy measures.